# α-synuclein overexpression and the microbiome shape the gut and brain metabolome in mice

**DOI:** 10.1038/s41531-024-00816-w

**Published:** 2024-10-30

**Authors:** Livia H. Morais, Joseph C. Boktor, Siamak MahmoudianDehkordi, Rima Kaddurah-Daouk, Sarkis K. Mazmanian

**Affiliations:** 1 Division of Biology and Biological Engineering, California Institute of Technology, Pasadena, CA USA; 2 Aligning Science Across Parkinson’s (ASAP) Collaborative Research Network, Chevy Chase, MD 20815 USA; 3 Department of Psychiatry and Behavioral Sciences, Duke University, Durham, NC USA; 4 Duke Institute of Brain Sciences, Duke University, Durham, NC USA; 5 Department of Medicine, Duke University, Durham, NC USA

**Keywords:** Parkinson's disease, Microbiology, Animal disease models, Metabolomics

## Abstract

Pathological forms of α-synuclein contribute to synucleinopathies, including Parkinson’s disease (PD). Most cases of PD arise from gene-environment interactions. Microbiome composition is altered in PD, and gut bacteria are causal to symptoms in animal models. We quantitatively profiled nearly 630 metabolites in the gut, plasma, and brain of α-synuclein-overexpressing (ASO) mice, compared to wild-type (WT) animals, and comparing germ-free (GF) to specific pathogen-free (SPF) animals (*n* = 5 WT-SPF; *n* = 6 ASO-SPF; *n* = 6 WT-GF; *n* = 6 ASO-GF). Many differentially expressed metabolites in ASO mice are also dysregulated in human PD patients, including amine oxides, bile acids and indoles. The microbial metabolite trimethylamine N-oxide (TMAO) strongly correlates from the gut to the plasma to the brain in mice, notable since TMAO is elevated in the blood and cerebrospinal fluid of PD patients. These findings uncover broad metabolomic changes that are influenced by the intersection of host genetics and microbiome in a mouse model of PD.

## Introduction

Parkinson’s disease (PD) is the second most prevalent neurodegenerative condition, affecting 3% of the elderly population^1^ and presenting a significant social and economic burden that is growing as lifespans increase^2^. The hallmark symptom of PD is progressive movement dysfunction, which can include tremors, stiffness, and difficulty with balance and coordination. Currently available treatments can be effective but often induce undesirable side effects, are difficult to dose, and are not disease-modifying. The etiology of PD is multifactorial, with both genetic and environmental factors contributing to pathophysiology^3,4^. Mutations in, or overexpression of, the *SNCA* gene which encodes the neuronal protein α-synuclein (αSyn) increase risk for developing PD^5^. In healthy neurons, αSyn regulates cellular homeostasis by modulating synaptic function and neurotransmitter release^6^. In pathological conditions, conformational changes in αSyn, phosphorylation at serine residue 129, and accumulation of aggregated forms are associated with synucleinopathies, a family of disorders with wide-ranging clinical presentations^7^, with PD being the most prevalent and best studied. Pathological species of αSyn can also be found outside the central nervous system (CNS). For example, phosphorylated αSyn is markedly elevated in the gastrointestinal (GI) tract of patients up to 20 years before PD diagnosis^8–10^, and many individuals with PD will experience clinical GI symptoms in the prodromal phase, with constipation being correlated to PD severity^11^. Braak et al. proposed that some forms of PD may originate in the gut and subsequently spread to the brain, potentially explaining why GI symptoms precede motor deficits^12^. In animal models, αSyn pathology can propagate via neurons from the gut to the brain^13,14^.

Alterations in the gut microbiome, known as dysbiosis, are observed in neurodegenerative disorders such as PD and their associated preclinical models^15–20^. The fecal microbiome in human PD patients, compared to matched controls, contains fewer anti-inflammatory bacteria (i.e., *Blautia, Coprococcus, Faecalibacterium*, and *Roseburia*), while pathogenic taxa (i.e., *Streptococcus*, *Enterococcus*, and *Actinomyces*) are increased in relative abundance^21–24^. Functional analysis of the gut microbiome using shotgun metagenomics suggests that microbial metabolism and downstream metabolic pathways are altered in PD. For instance, a systematic analysis of the gut microbiome of individuals with PD revealed impaired bacterial amino acid synthesis, increased levels of homocysteine, and decreased levels of glutamate and glutamine^25^. Bacterial folate biosynthesis is also decreased in PD patients compared to healthy individuals^26^. In addition, the gut microbiome can alter the metabolism and systemic availability of the main drug used in the treatment of PD, levodopa (L-dopa)^20,27,28^. Metabolomic surveys of various biological samples (i.e., cerebrospinal fluid (CSF), plasma, serum, sebum, saliva, and feces) have uncovered changes in the presence and levels of various amino acids, amines, urate, lipids, and other chemicals in PD^29–34^. While gut bacteria are significant modulators of the metabolite repertoire in humans, contributing an estimated 50% of the small molecules in blood^35^, whether and how PD is impacted by metabolomic dysregulation remain unknown.

In mice, overexpression of human αSyn from the Thy1 promoter models certain forms of PD that may result from gene duplication or increased gene expression. αSyn-overexpressing (ASO) mice exhibit progressive motor deficits, GI symptoms, and αSyn pathology in the gut and brain^36,37^. Our laboratory has revealed that ASO mice raised in germ-free conditions or treated with broad spectrum antibiotics, i.e., mice without a microbiome, do not develop motor dysfunction and do not show αSyn aggregates in the brain^19^. We also showed that fecal microbiota transplant (FMT) from human PD patients into ASO mice worsens motor deficits compared to FMT from healthy donors^19^. Interestingly, infection of PD mouse models with enteric pathogens or induction of intestinal inflammation worsens PD-like phenotypes^38–41^. Conversely, dietary interventions that restore healthy microbiome profiles ameliorate motor deficits and αSyn pathology in the substantia nigra and striatum of ASO mice^42^. The microbiome is also altered in non-human primate models of PD^43,44^, and numerous microbiome surveys in humans have shown stereotypical changes in the fecal microbiome between PD patients and household and population controls^45–48^. Collectively, these studies support the hypothesis that the microbiome is an environmental modifier of genetic risk in PD. Based on the microbiome’s profound impacts on metabolism, we performed targeted metabolomic profiling of ASO mice under standard laboratory or germ-free housing conditions. We report tissue-specific metabolite changes driven by genetics, the microbiome, or both that are reminiscent of metabolomic signatures in human PD. We also identify characteristic changes in microbial molecules that link the gut and the brain. These findings advance our understanding of gene-environment interactions associated with synucleinopathies such as PD.

## Results

### Metabolomic profiles in mice are shaped by αSyn overexpression and the microbiome

**Fig. 1:**
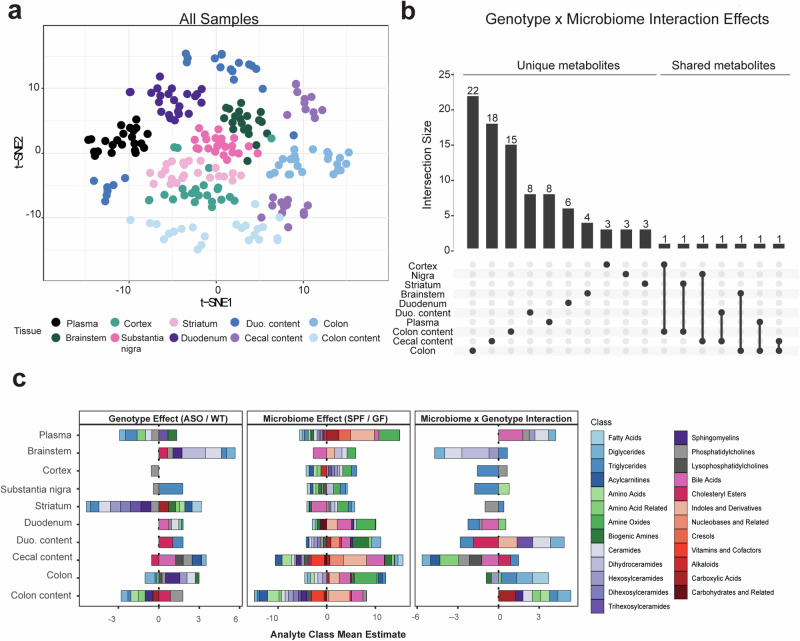
αSyn overexpression and microbiome presence alter global metabolomic profiles in mice. **a** tSNE plot of all metabolomic samples in this study, colored by tissue. **b** UpSet plot of unique and shared metabolite sets across all samples. Intersection size describes the number of metabolites with a significant genotype×microbiome interaction effect (*p* < 0.05). The dots below the bar chart indicate the sample source of the metabolites. Singular dots with no vertical lines connecting to other tissues indicate a set of metabolites which are uniquely altered in a particular tissue. **c** Stacked barplots depicting average effect sizes for biochemical classes containing molecules significantly associated (*p* < 0.05) with either a genotype, microbiome, or interaction effect in a linear regression model.

To explore the effects of genotype and microbiome on the metabolome in a PD mouse model, “Line 61” Thy1-ASO and wild-type (WT) littermates were reared in specific pathogen-free (SPF; standard laboratory microbiome) or germ-free (GF) conditions to 4 months of age, when ASO mice display robust motor symptoms, constipation-like phenotypes, oxidative stress, and mitochondrial alterations^19^. Samples were collected from segments of the gut, from the blood, and from various brain regions, and analyzed with the Biocrates MxP Quant 500 platform which measures 630 unique metabolites across 26 biochemical classes chosen to capture influences of diet and host-microbial interaction. Across all samples, we observed chemical features clustering by tissue (Fig. 1a), as expected.

We analyzed conditional genotype effects (WT vs. ASO) and/or microbiome (SPF vs. GF) effects on the metabolome. Metabolite profiles in SPF mice reflect the metabolic products of the microbiome, the host, and their co-metabolism. Due to sampling several tissue types and the experimental design profiling 4 groups of mice, few metabolites passed a false discovery rate (FDR) threshold of 0.1; instead, we present all findings with *p* values ≤ 0.05 (Supplementary Data). Across all tissues, we observed sets of metabolites significantly altered by genotype (Fig. S1a), microbiome (Fig. S1b), or the interaction between both (Fig. 1b). Individual metabolites displayed pronounced tissue specificity, with limited overlap between the gut and the brain (Fig. 1b). However, three metabolites impacted by the interaction between genotype and microbiome were shared between the gut and brain: triglyceride (TG) (16:0_40:7), diglyceride (DG) (14:1_18:1), and taurine. Additional molecules found in both tissues showed either genotype- or microbiome-specific changes (Fig. S1). We observed that genotype primarily influenced metabolites in the striatum, whereas the gut microbiome predominantly affected the metabolome in the colon, colonic contents, and cecal contents. Similarly, most of the analytes whose abundance was significantly influenced by the interaction of genotype and microbiome were altered in the gut (Fig. 1b).

To assess functional changes in the metabolome, we grouped significantly altered metabolites by biochemical features (Fig. 1c and Supplementary Data). We observed a broad range of molecular classes affected by the presence or absence of a microbiome, compared to more limited effects between ASO and WT animals. The microbiome influenced metabolite classes in all biological samples, with adjacent sites within gut or brain tissues displaying similar alterations (Fig. 1c). In SPF mice, we observed enrichment of metabolite classes synthesized and modulated by gut microbes, including amine oxides, bile acids, and indoles and their derivatives. In ASO mice, the most significant alterations occurred in lipid metabolites (Fig. 1c). At the molecular class level, there were minimal genotype-microbiome interactions.

### The gut microbiome modulates levels of bioactive metabolites in the gut of ASO mice

**Fig. 2:**
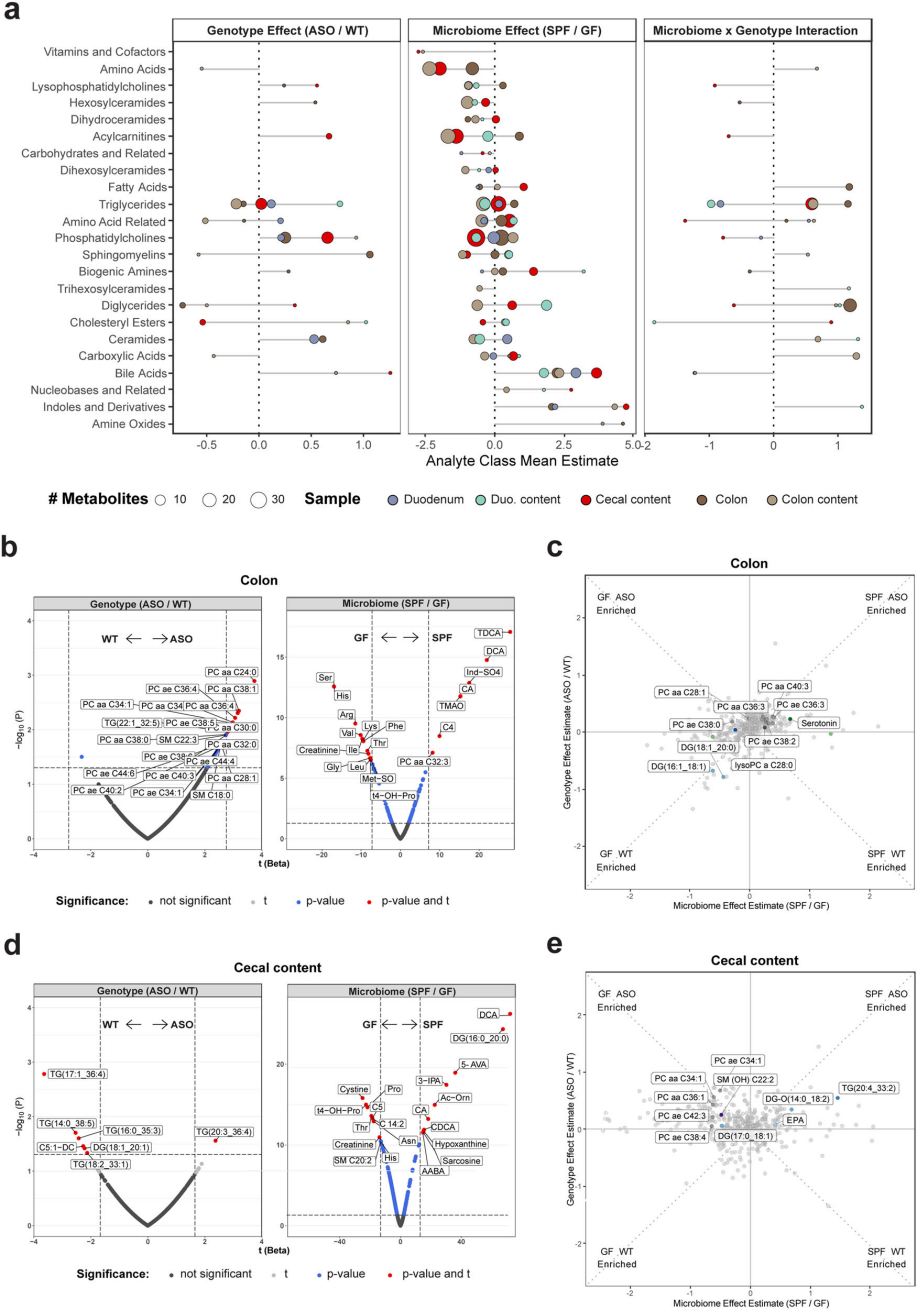
The gut microbiome shapes metabolism similarly across the GI tract. **a** Lollipop plot of relative enrichment/depletion of significantly altered (*p* < 0.05) metabolites in gut samples, organized by molecular class. Data points are colored by sample source and sized by number of metabolites. Volcano plots showing metabolites significantly enriched by genotype or microbiome status in the colon (**b**) and cecal contents (**d**). Scatterplots of metabolites in the colon (**c**) and cecal contents (**e**) affected by the microbiome and genotype in a linear model. Colored points indicate metabolites significantly (*p* ≤ 0.05) altered by genotype and/or microbiome. Labeled points indicate the top 10 metabolites with the most significant genotype×microbiome interaction effect.

Having broadly identified classes of metabolites influenced by αSyn overexpression and the microbiome, we next focused on metabolomic profiles in the gut, where we observed the most pronounced metabolite changes in response to microbiome status. Microbial communities vary spatially along the gastrointestinal (GI) tract, and accordingly we analyzed the metabolome in different regions of the small and large intestines. At the level of functional metabolite classes, we discovered that the microbiome induced highly concordant shifts across all gut tissues, while genotypic effects were more specific to different segments of the gut (Fig. 2a), suggesting that αSyn overexpression and/or aggregation is not uniform along the GI tract in this model.

Consistently altered metabolites in ASO vs. WT animals included TGs and DGs, as well as phosphatidylcholines (PC), the most abundant membrane phospholipid^49^. ASO mice displayed higher overall PC abundance in the colon (Fig. 2b, c), with elevated levels of PC aa C28:1 and PC ae C38:0 specifically in ASO-GF mice (Fig. 2c). A similar trend was evident in cecal contents, where PC aa C34:1 and PC aa C36:1 were more abundant in ASO-GF compared to ASO-SPF animals (Fig. 2d, e). In SPF vs. GF animals, we observed increased levels of nucleobases, and bile acids such as deoxycholic acid (DCA) and taurodeoxycholic acid (TDCA) (Figs. 2 and S2). We also discovered that levels of indoles and their derivatives, including indoxyl sulfate (Ind-SO_4_), were impacted by the microbiome (Figs. 2 and S2). Collectively, these findings reveal that microbial metabolites associated with signaling to the immune and nervous systems are dysregulated in the gut of ASO mice.

### The metabolome in PD-relevant brain regions is differentially affected by the microbiome

**Fig. 3:**
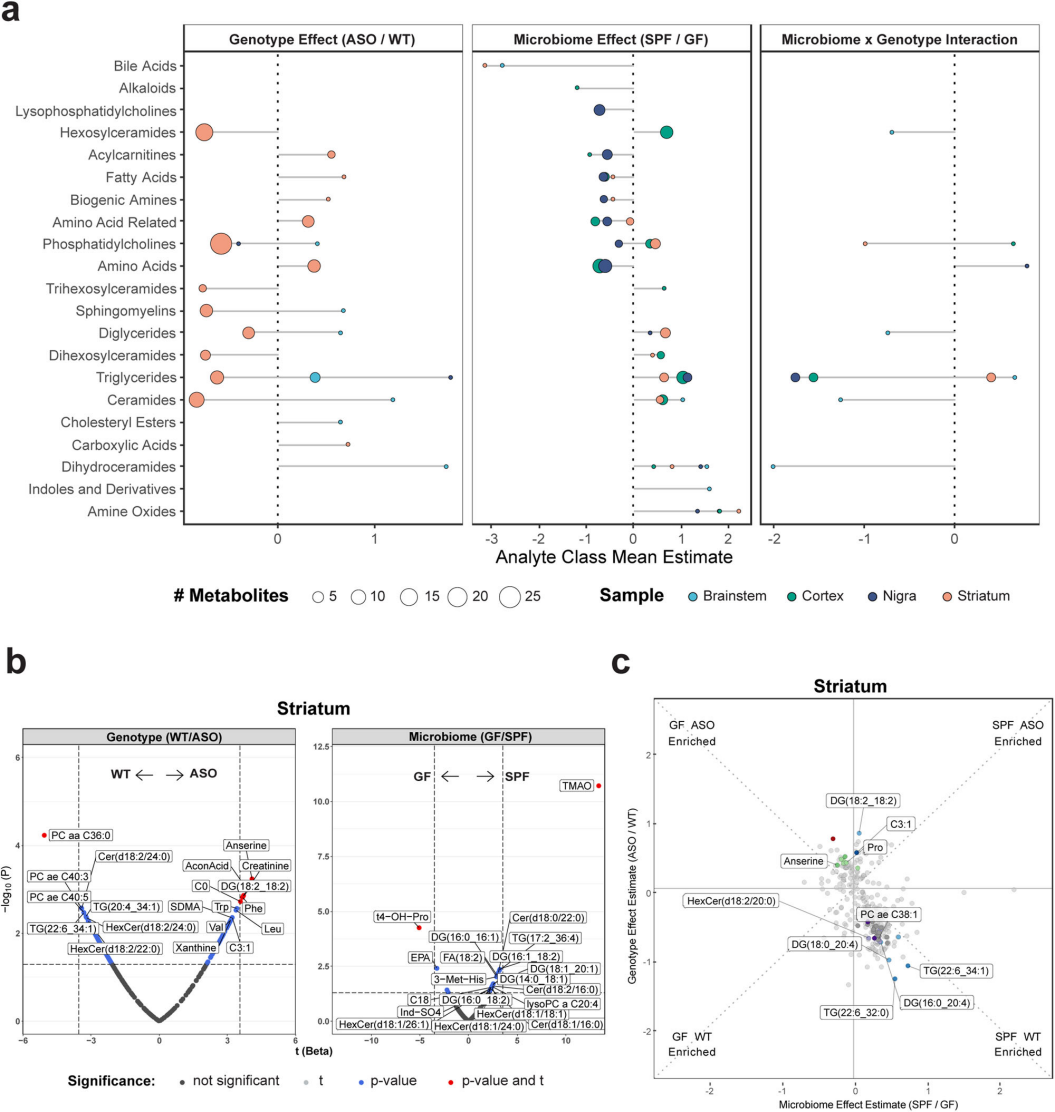
The genotype and microbiome alter metabolite levels differentially across the brain. **a** Lollipop plot of relative enrichment/depletion of metabolites whose levels are significantly altered (*p* < 0.05) by genotype, microbiome, or their interaction in the brain. Data points are colored by tissue and sized by number of metabolites. **b** Volcano plots showing metabolites significantly enriched by genotype or microbiome status in the striatum. **c** Scatterplot of metabolites in the striatum affected by the microbiome and genotype in a linear model. Colored points indicate metabolites significantly (*p* < 0.05) altered by genotype and/or microbiome. Labeled points indicate the top 10 metabolites with the most significant genotype×microbiome interaction effect.

We next explored genotype-microbiome interactions in shaping the brain metabolome. PD is primarily associated with the progressive loss of dopaminergic neurons in the substantia nigra that project to the striatum^50^. However, neurodegeneration and pathology also occur in other areas^50^, and accordingly we examined various brain regions, including the brainstem, cortex, substantia nigra, and striatum. We observed clustering of metabolomes within each brain tissue and less separation between brain tissues compared to samples from the gut (Fig. 1a), indicating a brain-specific metabolomic signature. Comparing ASO to WT animals, we report unique differences in the metabolome within the striatum (Fig. 3a), with a notable increase in neuroactive amino acids—anserine, creatinine, and aconitic acid (AconAcid) (Fig. 3b, c). Interestingly, anserine levels were further enriched in ASO-GF animals, and proline (Pro), another oxidative stress modulator^51^, was higher in ASO-SPF mice than in other animal groups (Fig. 3c). ASO mice also harbored higher levels of phenylalanine (Phe) and tryptophan (Trp), precursors of dopamine and serotonin, respectively, in the striatum (Fig. 3b). In the cortex, ASO mice contained elevated levels of 3-methylhistidine (3-Met-His) (Fig. S3c). ASO mice showed decreased abundance of several lipids, including ceramides, TGs, and PCs throughout the brain, but particularly in the striatum and cortex (Figs. 3b,c and S3). Interestingly, a unique converse effect was observed in the brainstem, with more TGs in ASO mice (Fig. S3a). The brainstem, which is innervated by the autonomic nervous system, serves as a central hub for lipid sensing^52^, suggesting potential systemic dysregulation of lipid metabolism in response to αSyn overexpression.

Changes in the metabolome were evident across all brain regions (Figs. 3 and S3). Regardless of genotype, the gut microbiome significantly shaped lipid metabolism, influencing key metabolites such as TGs, DGs, ceramides, and lysophosphatidylcholine (LysoPC), with notable enrichment of lipids in the brains of SPF animals (Figs. 3 and S3). The most dramatic influence of the microbiome throughout the brain was a significant increase in levels of trimethylamine N-oxide (TMAO) (Figs. 3b and S3).

### A single microbially-synthesized metabolite connects the GI tract, plasma, and the brain

**Fig. 4:**
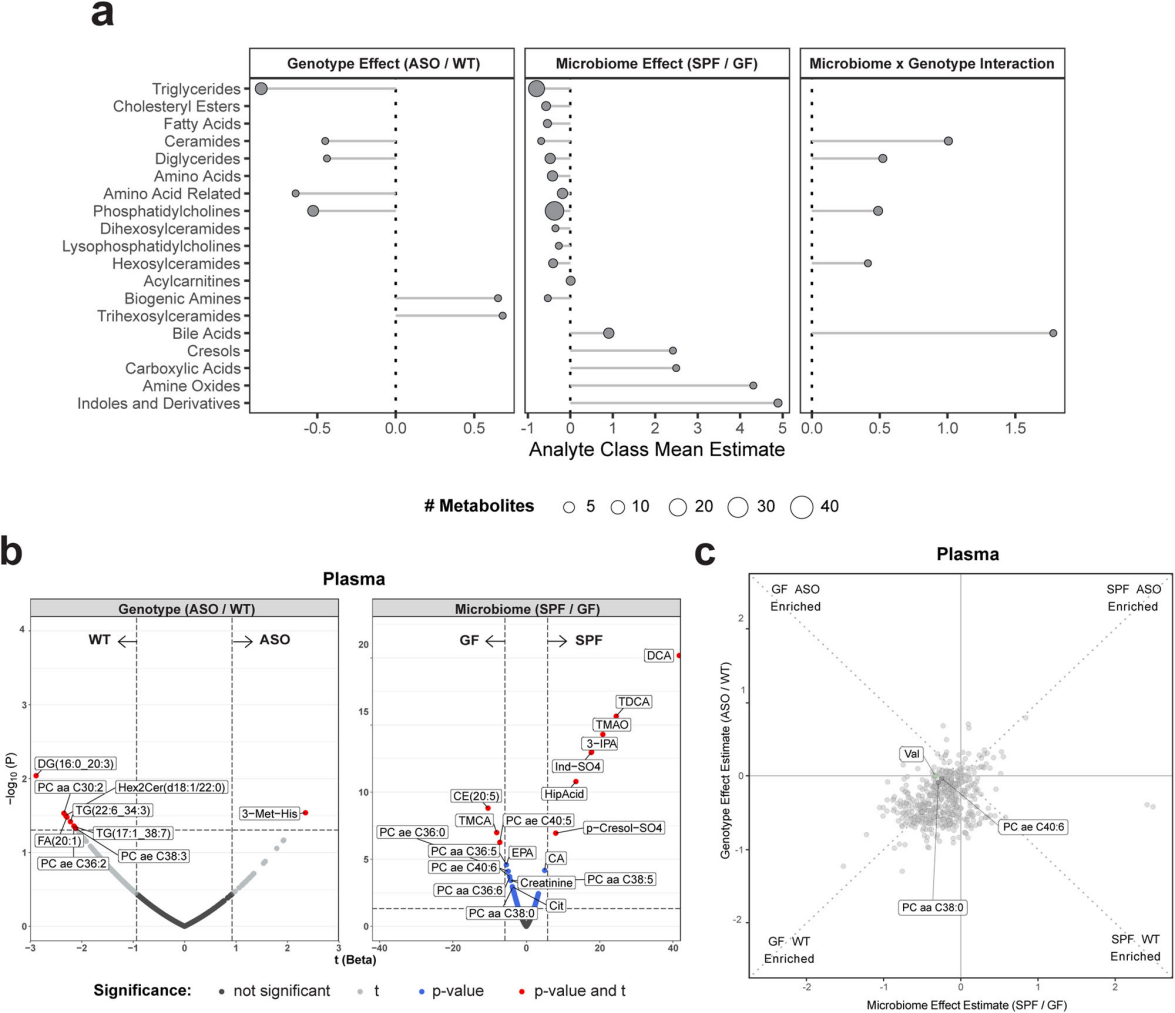
αSyn overexpression and microbiome influence circulating metabolites. **a** Lollipop plot of relative enrichment/depletion of metabolites whose levels are significantly altered (*p* < 0.05) by genotype, microbiome, or their interaction in plasma. Data points are colored by metabolite class and sized by number of metabolites. **b** Volcano plots showing metabolites significantly enriched by genotype or microbiome status in the plasma. **c** Scatterplot of metabolites in the plasma affected by the microbiome and genotype in a linear model. Colored points indicate metabolites significantly (*p* < 0.05) altered by genotype and/or microbiome. Labeled points indicate the top 10 metabolites with the most significant genotype×microbiome interaction effect.

To explore potential links between altered central and peripheral metabolism, we examined the plasma since metabolites may travel from the gut to the brain (and vice versa) through blood. In ASO animals, we observed moderate depletion of lipid metabolites (Fig. 4a), suggestive of dysregulation in lipid homeostasis, with DGs, TGs, and PCs being the most affected by the microbiome (Fig. 4b). Valine, a branched-chain amino acid (BCAA) involved in energy generation^53^, was enriched in GF animals compared to SPF counterparts, highlighting the influence of gut bacteria on amino acid metabolism (Fig. 4c).

**Fig. 5:**
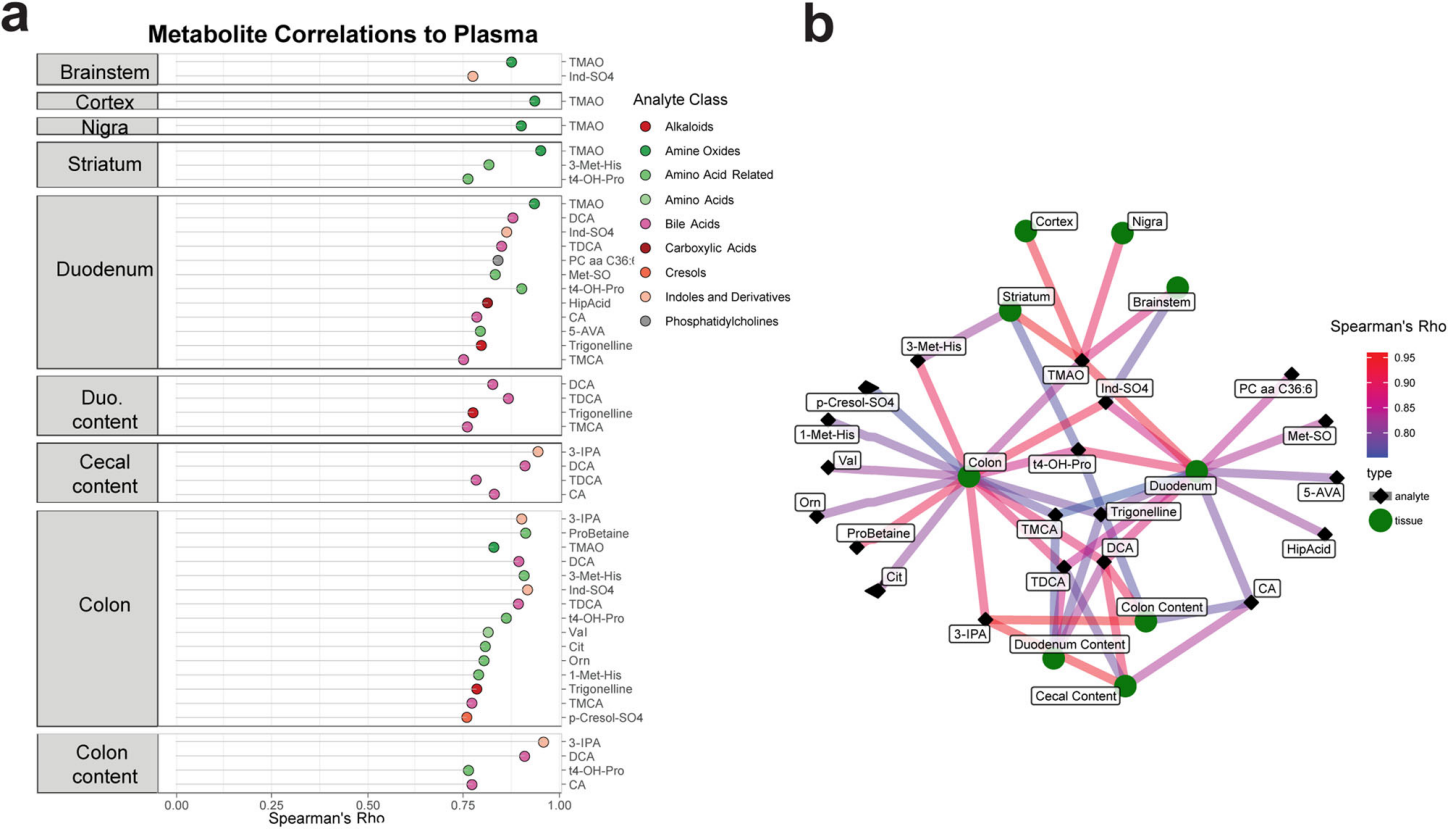
A microbially-produced metabolite links the gut-brain axis in ASO mice. **a** Lollipop plot showing metabolites whose levels in each indicated sample correlate strongly (Spearman’s Rho > 0.75) with the level of the same metabolite in plasma. **b** Network visualization of metabolites shown in **a**. A metabolite is connected to a tissue node if its abundance there is strongly correlated with its abundance in plasma. TMAO is the most highly connected metabolite, with strong correlations to plasma levels in six different tissue types.

When we correlated levels of metabolites detected in plasma with their levels in other tissues, molecular features with the strongest plasma level correlation were those linked to gut microbes (Fig. 5a). In the brain, these metabolites included Ind-SO_4_, amino acid-related compounds, and TMAO. The levels of many additional metabolites were strongly correlated between the plasma and gut tissues and contents, including bile acids, indoles and their derivatives, and amino acid-related molecules. TMAO was notable in being strongly correlated between the GI tract and plasma, as well as between plasma and brain tissues (Fig. 5b). Our study design, analyzing multiple sites from the gut to the brain, uncovers TMAO as a potentially pathogenic molecule that may link the gut microbiome to the brain in ASO mice.

## Discussion

αSyn overexpression (a genetic factor) and microbiome alterations (an environmental factor) have been implicated in PD. We reveal herein that gene-microbiome interactions shape the metabolome in a mouse model of synucleinopathy. Notably, many of the metabolites that were altered in ASO mice have been implicated in human PD, affecting mitochondrial function, oxidative stress response, inflammation, protein aggregation, and neurotransmission. At the timepoint we assessed, 4 months of age, ASO mice show motor symptoms, brain pathology, oxidative stress, and mitochondrial alterations, but do not yet display detectable neurodegeneration, thus modeling an early stage of human disease progression^37,50,54^. As the microbiome is altered in PD patients, and gut bacteria impact motor and non-motor symptoms in several PD models, dysregulation of microbial metabolites may represent a key aspect of PD pathophysiology that warrants further investigation.

Lipid homeostasis has been proposed as a key feature of PD^55^. We uncovered that both genotype and the microbiome influence lipid abundance, particularly of PCs in the brain and gut. Alterations in lipid metabolism have been widely implicated in PD and other neurodegenerative disorders, including Alzheimer’s disease^56,57^. Interestingly, PCs influence the aggregation of αSyn^58^. Brains of individuals with PD display decreased PC in the visual cortex^59^, and a similar reduction was seen in a rat model of early-stage PD^60^. In the gut, PC plays a significant role in the colonic mucosa, forming a hydrophobic barrier to protect from inflammatory insults^61^. Lower levels of PCs in the luminal mucus may contribute to gut mucosal inflammation and other GI issues such as constipation and delayed gastric emptying in PD^62^. In addition to reduced levels in the gut and brain, we observed moderate depletion of lipid metabolites, particularly PCs and TGs, in the plasma of ASO mice, suggesting widespread dysregulation of lipid homeostasis. In humans, an elevated PC/lysophosphatidylcholine (lyso-PC) ratio has been reported in the plasma of individuals with PD^63^. Similarly, lower levels of serum TGs, non-esterified fatty acids, and cholesterol are observed in the A53T human αSyn mouse model and correlate with weight loss^64^. In humans, lower serum and plasma levels of TGs and cholesterol are linked to PD^65–67^, and alterations in lipid metabolism have also been described in AD and other neurodegenerative conditions^68–70^. Taken together, alterations in lipid metabolism in the gut, brain, and plasma in ASO mice diplay similarities to lipid dysregulation in human PD.

In gut-derived samples, we found interesting profiles for microbially-derived metabolites implicated in inflammation. As expected, GF mice had lower levels of secondary bile acids and indole metabolites. Bile acids are produced in the liver and conjugated by certain gut bacteria into secondary bile acids such as DCA and TDCA, which regulate metabolic and signaling functions^71–73^. Altered bile acid profiles have been observed in the GI tracts of individuals with PD compared to controls^47,74^ as well as in αSyn-based mouse models^75^. Bile acids have diverse functions ranging from facilitating nutrient absorption to impacting immune responses in the gut and systemic compartments, and altered bile acid profiles in the brain have been associated with depression and Alzheimer’s disease (AD)^76–78^. Elevated levels of secondary bile acids such as DCA and lithocholic acid (LCA) are associated with the prodromal stage of PD and are linked to an increase in Clostridiales cluster XI in the gut^75^. Functional microbiome analysis and metabolic modeling has suggested that *Akkermansia muciniphila*, *Arcanobacterium ihumii*, *Alistipes shahii*, and *Candidatus* Gastranaerophilales increase production of indole and its derivatives such as indole*-*3*-*propionic acid (IPA), resulting in elevated levels in the serum of PD patients^26^. Another indole metabolite, Ind-SO_4,_ has been linked to increased oxidative stress^79^, a notable feature of PD pathology and the concentration of Ind-SO_4_ in the urine of PD patients is doubled compared to individuals without PD^80^. While we observed a microbiome effect on indole abundance, we did not see a genotype effect in ASO mice.

In the brain, changes in amino acid profiles suggest effects on oxidative stress and neurotransmission, as well as energy metabolism. We found that ASO mice exhibit higher levels of neuroactive amino acids with crucial roles in regulating oxidative stress and redox homeostasis^81^. A recent study in the αSyn A53T mouse model of PD also observed accumulation of carnosine, homocarnosine, and anserine in the brain, which was theorized could be a defense against reactive oxygen species (ROS)^82^. Interestingly, we reveal that anserine levels are further enriched in ASO-GF animals, indicating a combinatorial effect of genotype and the microbiome. Anserine and carnosine abundances are decreased in cortex samples from humans with PD^83^. Anserine is also reduced in the CSF of PD patients^84^. These discrepancies may reflect our analysis at a pre-neurodegeneration stage (4 months of age) when compensatory and neuroprotective mechanisms have been observed in response to accumulation of αSyn^85,86^. αSyn overexpression in mice also increased levels of proline, an oxidative stress modulator^51^ that is increased in the serum of humans with PD^87^. Levels of 4-hydroxyproline are also increased in CSF^88^. AconAcid is a fatty acid in the tricarboxylic acid (TCA) cycle which also influences oxidative phosphorylation. In individuals with PD, the levels of AconAcid and other TCA cycle metabolites are reduced in the blood^89,90^; therefore it would be interesting to measure these molecules longitudinally in human brain and CSF. Levels of Phe and Trp, precursors for dopamine and serotonin, respectively, were increased in the striatum of ASO mice. These neurotransmitter pathways are impacted in PD^91,92^, and our observations align with metabolomic profiling of CSF from PD patients showing that Phe and Trp accumulation correlates with disease progression^93^. Interestingly, among the brain regions profiled in our study, the striatum was the most metabolically imprinted by both genotype and the microbiome.

In the plasma, we uncovered elevated levels of valine, a BCAA, in ASO mice lacking a microbiome. A recent study identified changes in BCAA concentration in the serum of individuals with PD that correlated with disease stage and gut microbiome composition^65^. Furthermore, serum BCAA concentrations are lower in individuals with advanced-stage PD^65^. In dopaminergic neurons derived from stem cells from human PD patients, the relative abundance of valine is also diminished and is linked to disrupted mitochondria-lysosome contact dynamics^94^.

We modeled the flux of metabolites from the gut to the brain via plasma, and discovered a strong signal for the microbial metabolite TMAO. Trimethylamine (TMA) is produced by gut bacteria through conversion of dietary precursors such as choline, betaine, and L-carnitine, followed by conversion into TMAO in the liver^95^. TMAO has been associated with infiltration of inflammatory cells in the colon and rectal epithelium, as well as cellular damage^96^. TMAO is strongly implicated in arteriosclerotic cardiovascular disorders and systemic inflammation^97^. Importantly, levels of TMAO and related metabolites are increased in CSF and plasma from PD patients^98^. The role of TMAO in the CNS remains largely unclear, with both beneficial and detrimental effects reported^99,100^. TMAO is associated with brain inflammation, astrocyte activation, and cognitive deficits in mice^101^, as well as neuronal senescence and mitochondrial dysfunction^99^. In SPF animals, we observed elevated TMAO levels in various tissue types. TMAO levels are increased in the plasma and CSF of PD patients relative to controls^102,103^ and gene families involved in TMA production are enriched in the PD gut microbiome^24^, though not in all studies^104,105^. In individuals with PD, increased levels of TMAO and other bacterial-derived metabolites in the brain have been implicated in cognitive decline^83^, and higher levels of TMAO in the serum and CSF correlate with disease severity and the progression of motor symptoms^106^. TMAO has been shown to induce fibrillar aggregation of αSyn in a concentration-dependent manner in vitro^107–109^. Our findings add to growing evidence supporting a potential role for TMAO in PD.

The metabolome serves as a comprehensive indicator of environmental influences, including contributions of diet, toxins, drugs, and gut microbiome. Herein, we reveal broad changes in the metabolomic profile in gut, plasma, and brain of a mouse model of αSyn overexpression. Our study is limited by sample size, use of a single mouse model, and exclusion of sex as a variable due to the necessity of performing experiments exclusively in male ASO mice. However, our use of GF mice allows unequivocal assignment of metabolites that are regulated or produced by the gut microbiota, which is not possible in human studies. Most of the metabolomic surveys conducted in humans have used blood, urine and CSF samples, with only a few reports on brain and fecal samples^29–34^. Therefore, only limited direct comparisons can be currently made between the brain and gut metabolomes of mice with those reported from human tissue. Our discovery of changes in specific microbial molecules across multiple tissues in ASO mice, many of which correlate to findings in human studies, highlights the need for future investigations into the mechanistic role of gut bacteria in the pathophysiology of PD and other synucleinopathies.

## Methods

### Mice

Male mice overexpressing human αSyn under the Thy1 promoter (“Line 61” Thy1-α-Synuclein, ASO) and WT mice were generated by crossing wild-type BDF1 males (Charles River, RRID:IMSR_CRL:099) with ASO heterozygous females^37^. Since the transgene is carried on the X chromosome, only male animals were used to avoid the effects of X-inactivation, standard practice in studies with this mouse model^37,110–113^. This study used the following numbers of mice: *n* = 5 WT-SPF (2 litters); *n* = 6 ASO-SPF (3 litters); *n* = 6 WT-GF (4 litters); *n* = 6 ASO-GF (4 litters). GF mice were generated by caesarean section, with the offspring fostered by GF Swiss-Webster dams and maintained microbiologically sterile inside flexible film isolators^14,19,114^. SPF mice were housed in autoclaved micro-isolator cages. Mice were housed by litter (i.e., littermates of both genotypes were co-housed throughout the study). GF status was confirmed on a bi-weekly basis through 16S rRNA PCR of fecal-derived DNA and plating of fecal pellets on Brucella blood agar under anaerobic conditions and tryptic soy blood agar under aerobic conditions. Mice received food and water ad libitum, were maintained on the same 12-hour light-dark cycle and housed in the same facility. All animal husbandry and experiments were approved by the California Institute of Technology’s Institutional Animal Care and Use Committee (IACUC).

### Tissue dissection

At 4 months of age, mice were sacrificed by decapitation and samples collected from colon tissue, colonic contents, cecal contents, duodenum, duodenal contents, plasma, and brain. The brain was rapidly removed and placed in an ice-chilled stainless steel coronal matrix. Brain tissue was sectioned in slices of approximately 1 mm. Substantia nigra, striatum, motor cortex (referred to as cortex) and caudal brainstem (referred to as brainstem) were dissected within three minutes using reference brain atlas coordinates^115^. Gut tissue and contents were dissected on an ice-chilled stainless steel dissection tray. All samples were weighed, snap-frozen in dry ice and stored at −80 °C until processing. For protocol see 10.17504/protocols.io.14egn3pkzl5d/v3.

### Plasma collection

Trunk blood was collected in EDTA-coated tubes and kept at room temperature before plasma separation. Plasma was separated by centrifugation at 2500 × g for 10 minutes. Plasma was transferred to a pre-cooled collection vial and stored at −80 °C until processing. For protocol see 10.17504/protocols.io.14egn3pkzl5d/v3.

### Quantitative targeted metabolomics

#### Sample Preparation

Samples were prepared using the MxP Quant 500 kit (Biocrates life sciences AG, Innsbruck, Austria) in strict accordance with the manufacturer’s protocol. Plasma samples were centrifuged, and the supernatant was used for analysis. Brain, colon, and duodenum tissue samples were first suspended in 3 μL ethanol/phosphate buffer per mg tissue wet weight. The samples were then sonicated, vortexed and homogenized using a Precellys-24 instrument (Bertin Technologies, Montigny le Bretonneux, France), and the supernatant was used for analysis. For measurement of some metabolites, it was necessary to dilute the duodenum tissue samples 1:5 in buffer before the samples were centrifuged and the supernatant used for analysis. To extract metabolites from duodenal, cecal and colonic contents, samples were resuspended in extraction buffer (85% ethanol in phosphate buffer) and vortexed thoroughly until dissolved. After homogenization, the samples were ultrasonicated in a chilled bath for 5 min. Samples were then centrifuged and the supernatant was used for analysis. An additional 1:1000 dilution was prepared for the analysis of highly concentrated bile acids. For protocol see: 10.17504/protocols.io.261ge5pwyg47/v3.

#### Metabolite measurement

A mass spectrometry (MS)-based targeted metabolomics approach was used to determine the concentration of endogenous metabolites in a total of 226 samples: 23 plasma samples, 69 gut content samples (23 cecal contents, 23 colon contents, and 23 duodenum contents), and 134 tissue samples (88 brain, 23 colon, and 23 duodenum). Metabolites were quantified using the commercially available MxP^®^ Quant 500 kit (Biocrates). The kit provides measurements of up to 634 metabolites across 26 biochemical classes. Lipids (e.g, acylcarnitines, glycerophospholipids, sphingolipids, triglycerides) and hexoses were measured by flow injection analysis-tandem MS (FIA-MS/MS) using a 5500 QTRAP® instrument (AB Sciex, Darmstadt, Germany) with an electrospray ionization (ESI) source for the plasma and tissue samples, and a Xevo TQ-S (Waters, Vienna, Austria) instrument with an ESI source for the gut content samples. Small molecules were measured by liquid chromatography-tandem MS (LC-MS/MS), also using a 5500 QTRAP® instrument for all samples. Gut tissue and content samples were also measured by LC-MS/MS on a Xevo TQ-S instrument. To quantitatively analyze metabolite profiles in the samples, a 96-well-based sample preparation device was used which consists of inserts that have been impregnated with internal molecule standards labeled with heavy isotopes. A predefined sample amount was added to the inserts. Next, a phenyl isothiocyanate (PITC) solution was added to derivatize some of the analytes (e.g., amino acids), and after the derivatization was complete, the target analytes were extracted with an organic solvent, followed by a dilution step. The obtained extracts were then analyzed by FIA-MS/MS and LC-MS/MS methods using multiple reaction monitoring (MRM) to detect the analytes. Data were quantified using appropriate mass spectrometry software, either Sciex Analyst® (https://sciex.com/, RRID:SCR_023651) or Waters MassLynx™, https://www.waters.com/waters/en_US/MassLynx-Mass-Spectrometry-Software-/nav.htm?cid=513164, RRID:SCR_014271), and imported into Biocrates MetIDQ™ software for further analysis. The accuracy of the metabolite quantification was determined by comparing to human plasma-based quality control samples that were included in every plate. For protocol see: 10.17504/protocols.io.261ge5pwyg47/v3.

#### Quality control

The raw Q500 metabolomic profiles included measurements of 634 metabolites in 226 samples. Quality control steps were performed as in prior publications^116,117^. Separately for each material type, metabolites with >30% of measurements above the lower limit of detection (LOD) in SPF animals were included (*n* = 539, 459, 503, and 297 remaining metabolites in plasma, GI tissue, gut content, and brain tissue, respectively). Imputation of <LOD values was performed using each metabolite’s LOD/2 value to increase statistical power. Since all samples for each material type were measured on one plate, no batch effect removal procedure was conducted. Metabolite concentrations were log_2_ transformed to achieve normal distribution.

### Statistical analysis

All statistical analyses were conducted in R 4.2.2 (https://www.r-project.org/, RRID:SCR_001905). We applied linear regression analysis incorporating covariates to correct for body weight, and t-distributed stochastic neighbor embedding (t-SNE) to reduce the dimensionality of metabolomic profiles for optimal visualization. t-SNE scatterplots were generated of log_2_-transformed metabolite concentrations using the Rtsne (v0.1-3.1, https://github.com/jkrijthe/Rtsne, RRID:SCR_016342) package with default parameters. To evaluate the overall effect of genotype, microbiome and their interaction on metabolome in each sample type, Permutational Multivariate Analysis of Variance (PERMANOVA) was conducted using the “adonis2” function in the vegan package (http://cran.r-project.org/web/packages/vegan/index.html, RRID:SCR_011950) with 10,000 permutations and Euclidean distance. Multivariate homogeneity of group dispersions was tested using the “betadisper” and “permutest” functions in the vegan package. To investigate the effects of genotype and microbiome status on the metabolome, we employed linear regression modeling that defined metabolites of interest that met either of the following criteria: 1) altered between ASO-SPF and WT-SPF animals, reflecting a genotype effect; and/or 2) altered between ASO-SPF and ASO-GF animals, reflecting a microbiome effect. Separate linear regression models were constructed for each metabolite within a tissue type. These models defined log_2_ metabolite concentration as the dependent variable, and body weight, microbiome, genotype and their interaction as independent variables. We did not account for potential litter effects. This allowed us to estimate the effects of genotype and microbiome status while controlling for variability in body weight. The linear model was specified as: (log_2_(metabolite [µM]) ~ Genotype + Microbiome + Genotype*Microbiome + Body-weight). For a non-parametric measure of metabolite-metabolite associations, we performed Spearman’s rank-based correlation.

## Supplementary information

Supplementary Information

Supplementary Data

## Data Availability

The datasets generated and analyzed in this study are available from: 10.5281/zenodo.10841426.

## Code availability

The underlying code for this study is available in GitHub and can be accessed via 10.5281/zenodo.10841426.
