# Supplementary material for: α-synuclein overexpression and the microbiome shape the gut and brain metabolome in mice
Source: NPJ Parkinsons Dis. 2024 Oct 30;10:208. doi: 10.1038/s41531-024-00816-w (PMC11525669; doi:10.1038/s41531-024-00816-w)
Supplement: Supplementary file 1 — Supplementary Information [file 41531_2024_816_MOESM1_ESM.pdf]

Supplementary Information

for

α-Synuclein Overexpression and the Microbiome Shape the Gut and Brain Metabolome in Mice

Livia H. Morais[#], Joseph C. Boktor[#], Siamak MahmoudianDehkordi[#], Rima Kaddurah-Daouk[*], Sarkis K. Mazmanian[*]

Contents:

- Supplementary Figures 1-3
- Legend for Supplementary Data

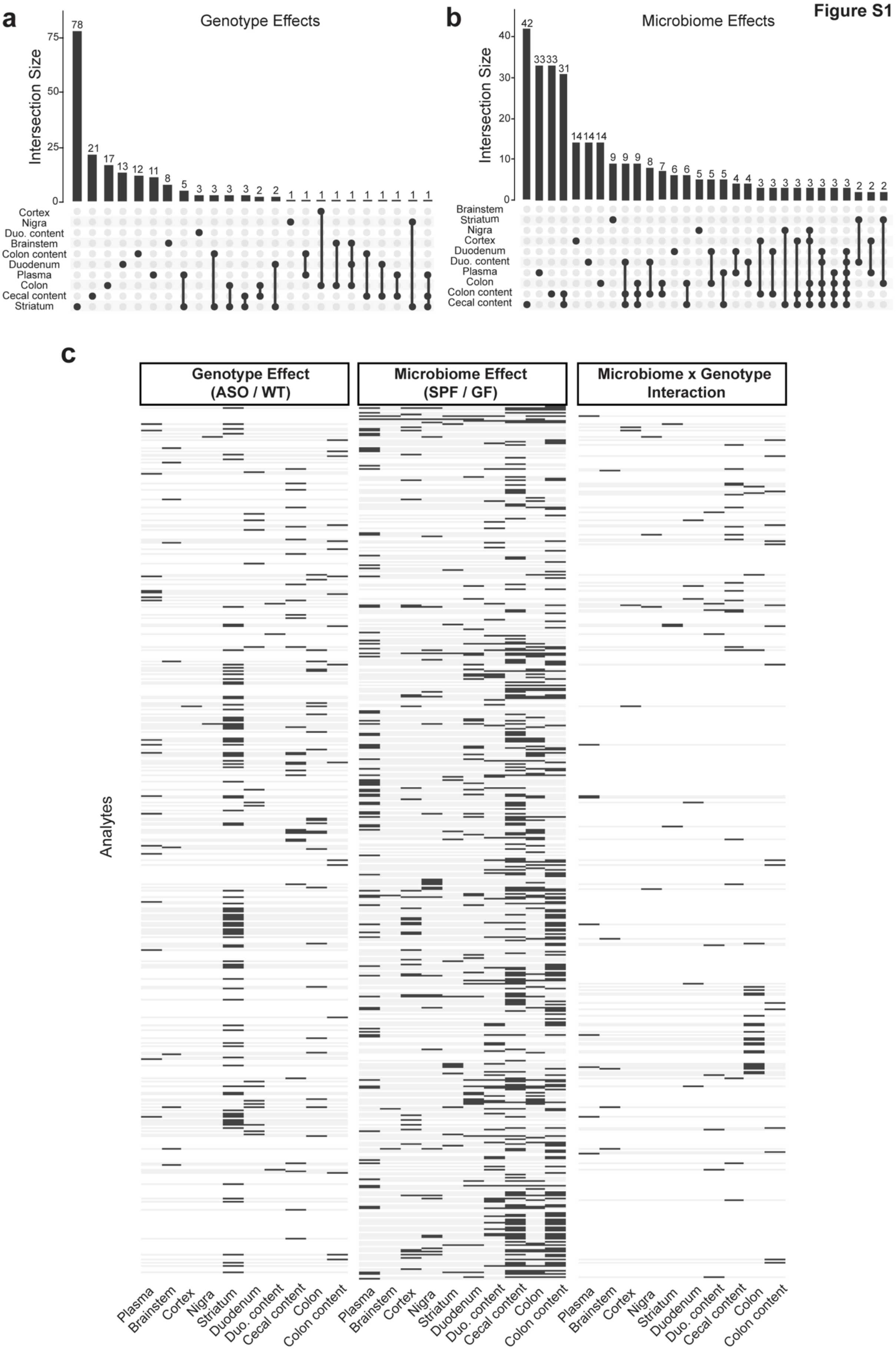
Figure S1
a
Genotype Effects
Intersection Size
78
21
17
13
12
11
8
5
3
3
3
3
2
2
1
1
1
1
1
1
1
1
1
1
Cortex
Nigra
Duo. content
Brainstem
Colon content
Duodenum
Plasma
Colon
Cecal content
Striatum
b
Microbiome Effects
Intersection Size
42
33
33
31
14
14
14
9
9
9
8
7
6
6
5
5
5
4
4
3
3
3
3
3
3
3
3
2
2
2
Brainstem
Striatum
Nigra
Cortex
Duodenum
Duo. content
Plasma
Colon
Colon content
Cecal content
c
Genotype Effect (ASO / WT)
Microbiome Effect (SPF / GF)
Microbiome x Genotype Interaction
Analytes
Plasma
Brainstem
Cortex
Nigra
Striatum
Duodenum
Duo. content
Cecal content
Colon
Colon content

Fig. S1: a-b) UpSet plots of unique and shared metabolite sets across all samples. Interaction size describes the number of metabolites with a significant genotype (**a**) or microbiome (**b**) interaction effect ($p<0.05$). The dots below the bar chart indicate the tissue source of the metabolites. Singular points with no vertical lines connecting to other tissues indicate a set of metabolites which are uniquely altered in a particular tissue. **c**) Binary heatmap of metabolites significantly altered in at least one tissue for at least one variable (genotype, microbiome, microbiome×genotype) from the linear model.

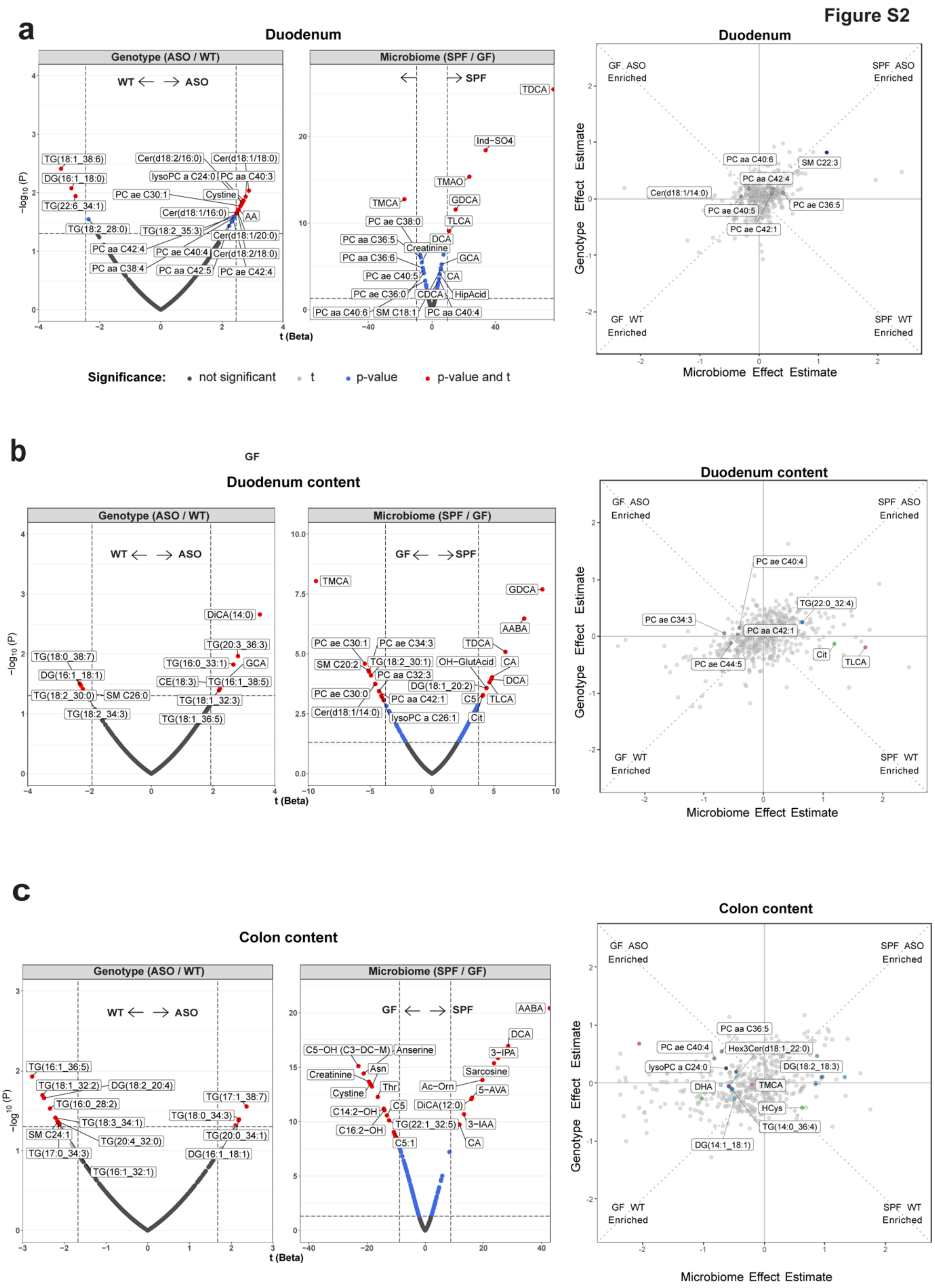

Fig. S2: Volcano plots and scatterplots of the most significantly altered metabolites showing genotype, microbiome, or genotype×microbiome interaction effects in the duodenum (**a**), duodenal contents (**b**), or colonic contents (**c**). Conventions are as in Fig. 2.

Figure S3

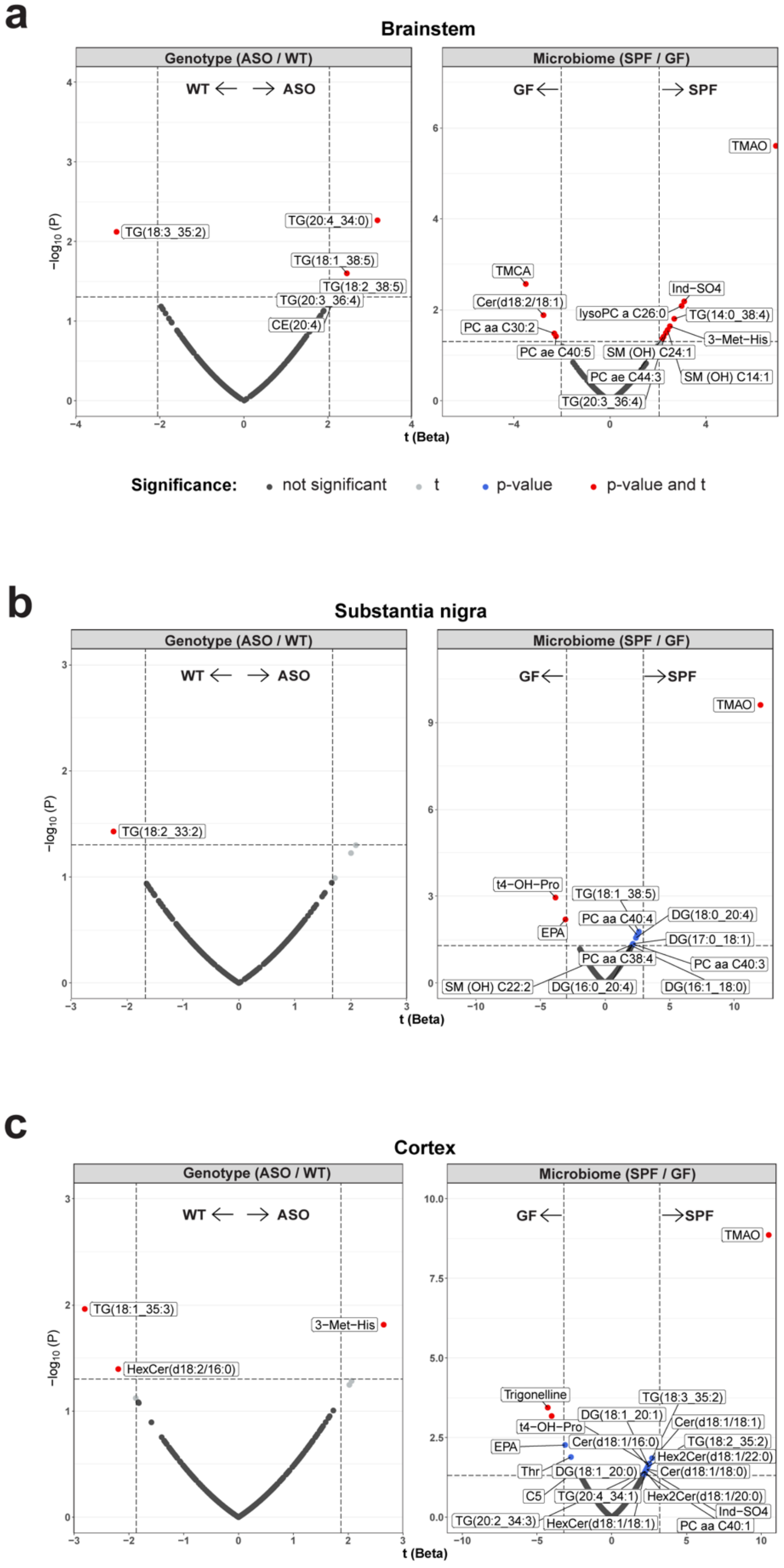

Fig. S3: Volcano plots of metabolites showing genotype or microbiome effects in the brainstem (**a**), substantia nigra (**b**) and cortex (**c**).

Supplementary Data:

Estimated log_2 fold differences in concentrations across different tissues for metabolites showing conditional effects of microbiome status or genotype. Estimated values were obtained from linear regression models adjusted for body weight.
